# Advances in Biological Wastewater Treatment Processes: Focus on Low-Carbon Energy and Resource Recovery in Biorefinery Context

**DOI:** 10.3390/bioengineering11030281

**Published:** 2024-03-16

**Authors:** J. Shanthi Sravan, Leonidas Matsakas, Omprakash Sarkar

**Affiliations:** 1 Research Center for Innovative Energy and Carbon Optimized Synthesis for Chemicals (Inn-ECOSysChem), Gwangju Institute of Science and Technology, Gwangju 61005, Republic of Korea; 2 Biochemical Process Engineering, Division of Chemical Engineering, Department of Civil, Environmental and Natural Resources Engineering, Luleå University of Technology, 971-87 Luleå, Sweden; leonidas.matsakas@ltu.se

**Keywords:** waste biorefinery, bioelectrochemical treatment, resource recovery, low carbon, circular economy, sustainable development goals

## Abstract

Advancements in biological wastewater treatment with sustainable and circularity approaches have a wide scope of application. Biological wastewater treatment is widely used to remove/recover organic pollutants and nutrients from a diverse wastewater spectrum. However, conventional biological processes face challenges, such as low efficiency, high energy consumption, and the generation of excess sludge. To overcome these limitations, integrated strategies that combine biological treatment with other physical, chemical, or biological methods have been developed and applied in recent years. This review emphasizes the recent advances in integrated strategies for biological wastewater treatment, focusing on their mechanisms, benefits, challenges, and prospects. The review also discusses the potential applications of integrated strategies for diverse wastewater treatment towards green energy and resource recovery, along with low-carbon fuel production. Biological treatment methods, viz., bioremediation, electro-coagulation, electro-flocculation, electro-Fenton, advanced oxidation, electro-oxidation, bioelectrochemical systems, and photo-remediation, are summarized with respect to non-genetically modified metabolic reactions. Different conducting materials (CMs) play a significant role in mass/charge transfer metabolic processes and aid in enhancing fermentation rates. Carbon, metal, and nano-based CMs hybridization in different processes provide favorable conditions to the fermentative biocatalyst and trigger their activity towards overcoming the limitations of the conventional process. The emerging field of nanotechnology provides novel additional opportunities to surmount the constraints of conventional process for enhanced waste remediation and resource valorization. Holistically, integrated strategies are promising alternatives for improving the efficiency and effectiveness of biological wastewater treatment while also contributing to the circular economy and environmental protection.

## 1. Introduction

Biological wastewater treatment strategies can contribute to environmental sustainability and green energy by reducing the environmental impact of wastewater discharge, recovering valuable resources from wastewater, and producing renewable energy and bioproducts [1,2]. However, there are also some challenges and limitations that need to be addressed, such as the high capital and operational costs, low stable performance and scalability of the reactors, quality and safety of the products, and regulatory and social barriers. Biological syntropy involves mutual synergism between the metabolic activities of microorganisms that cannot individually catabolize their metabolites [3,4]. Interspecies electron transfer (IET) is a kind of syntrophic mechanism wherein free electrons flow from one cell to another through shared physical and electrical connections [5,6]. The bioprocess efficiency is interlinked with the biocatalyst nature and sensitivity, substrate composition, low productivity/yields, byproduct formation, feedback inhibition, product purity, etc. [7,8]. Metabolic limitations can be addressed by syntrophic regulation with microorganisms or materials or electrodes. When the electrode serves as the electron acceptor (intermediate), an oxidized product is formed in the process referred to as unbalanced microbial metabolism [9,10,11].

Recalcitrant and emerging pollutants (persistent organic pollutants (POPs), nanoparticles, endocrine disruptors) and contaminants (industrial, hospital, domestic, and agricultural discharges and landfill leachate) are usually unregulated in terms of environmental discharge, having potential risks to human health and the environment, and need critical treatment [12]. These pollutants can enter wastewater streams from various sources, such as industrial discharges, agricultural runoff, hospital effluents, and domestic sewage. Emerging pollutants can disrupt biological treatment processes by inhibiting or killing microorganisms, altering microbial metabolism, or producing toxic intermediates [12]. Therefore, it is important to develop effective strategies to remove or degrade emerging pollutants in biological wastewater treatment systems. Conventional bioprocesses have limitations in the complete treatment of wastewater and hence require advanced integrated biological/bioelectrochemical remediation systems. Advanced oxidation processes (AOPs) are one of the most promising strategies for the removal of emerging pollutants in wastewater [13]. AOPs can generate highly reactive species, such as hydroxyl radicals, that can oxidize and degrade a wide range of organic compounds. Advanced oxidation processes can also improve the disinfection, color removal, and odor control of the wastewater [14]. The reaction mechanisms and process parameters of AOPs need critical consideration for effective application feasibility [15]. Electro-/bioelectro-/photo-chemical processes could also be effective in advanced wastewater treatment for recalcitrant/emerging pollutant removal. The integration of diverse biological treatment strategies with a broad scope of their individual advantages in a sequential and closed-loop pattern could be a sustainable approach [16,17]. These integrated biological/electrochemical/bioelectrochemical wastewater treatments can also benefit in energy and resource recovery, along with low-carbon generation [6,18]. Resources such as nitrogen, phosphorus, and sulfur can be removed from wastewater by biological processes, such as nitrification, denitrification, phosphorus accumulation, and sulfate reduction [19,20]. These nutrients can be recovered as valuable products, such as fertilizers, bioplastics, or chemicals, for agricultural or industrial applications. Energy can be recovered from wastewater by anaerobic processes, such as anaerobic digestion, dark fermentation (which involves hydrolysis, acidogenesis, acetogenesis, and methanogenesis), or microbial fuel cells [21,22,23].

These processes can convert the organic matter in wastewater into biogas, hydrogen, or electricity, which can be used for heating, lighting, or powering treatment plants or other facilities [24,25]. Nanotechnology can offer novel materials, such as nanoparticles, nanofibers, or nanocomposites, that can enhance biological treatment processes by providing catalysts, carriers, sensors, or antimicrobials [26,27,28,29]. Integrated biological wastewater treatment strategies can efficiently degrade organic pollutants in wastewater while also recovering valuable resources, such as energy, nutrients, minerals, and salts [16,17,30]. These strategies aim to reduce the environmental impact and operational cost of wastewater treatment, as well as to contribute to the circular economy and carbon neutrality goals [16].

**Figure 1:**
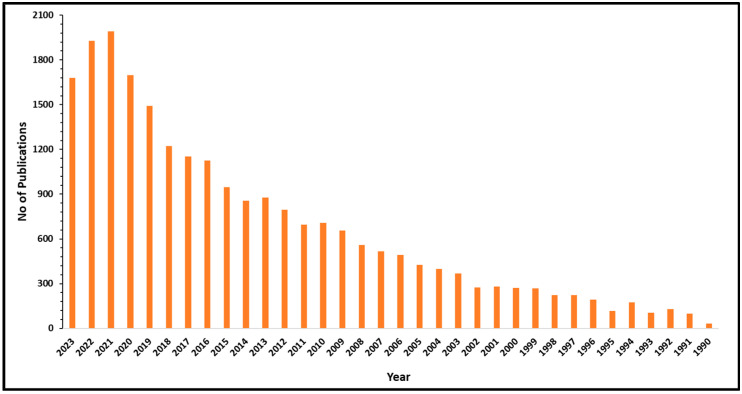
Histogram showing advanced biological wastewater treatment methods (selected keywords: bioremediation, electro-coagulation, electro-flocculation, electro-Fenton, advanced oxidation, electro-oxidation, bioelectrochemical systems) (source: Web of Science).

This review elaborates on advances in waste/wastewater treatment to address emerging pollutants toward energy and resource recovery with low-carbon outputs. Biological treatment methods, viz., bioremediation, electro-coagulation, electro-flocculation, electro-Fenton, advanced oxidation, electro-oxidation, bioelectrochemical systems, and photo-remediation, are summarized with respect to non-genetically modified metabolic reactions (Figure 1). Specific emphasis is placed on the challenges and limitations of advanced treatment processes that must be addressed to increase their energetic and economic applicability. Sustainable integrations with other treatment processes that could align with the circular economy perspective in a biorefinery approach are included.

## 2. Description of Conventional Methods for Wastewater Treatment

**Figure 2:**
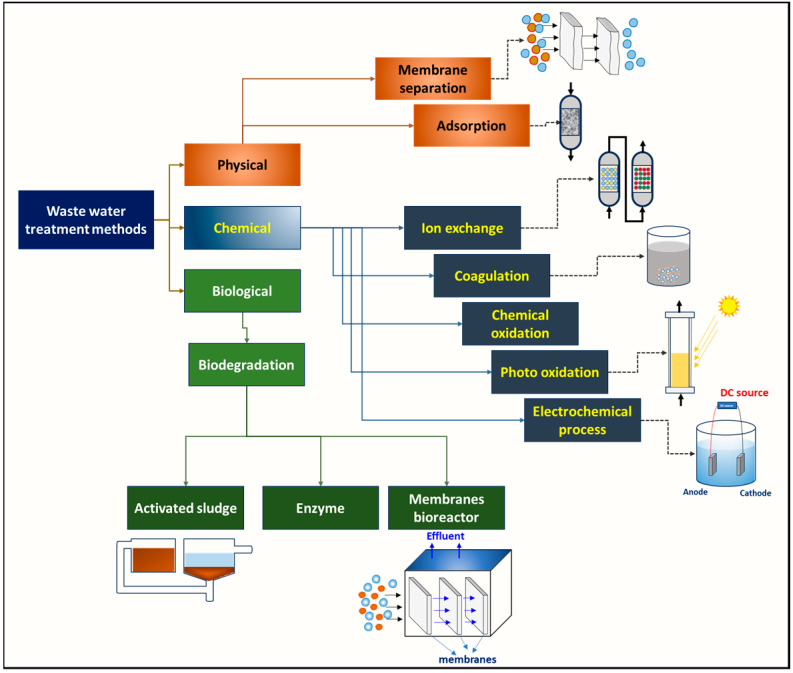
Schematic representing types of wastewater treatment methods.

Conventional biological wastewater treatment is widely used for domestic, municipal, and industrial wastewater treatments for organic pollutant and nutrient degradation [12,31]. Biological wastewater treatment is divided into aerobic and anaerobic processes. Aerobic treatment requires oxygen for the microorganisms to breakdown organic matter, whereas anaerobic treatment functions in oxygen-deprived conditions. Aerobic treatment is more common and can be further classified into suspended growth systems (such as activated sludge and oxidation ponds) and attached growth systems (such as trickling filters and rotating biological contactors). Anaerobic treatment is mainly used for high-strength wastewater or sludge digestion and produces biogas as a byproduct [32]. Anaerobic treatment can be performed in anaerobic lagoons or anaerobic bioreactors (such as up-flow anaerobic sludge blanket and anaerobic baffled reactors). The treatment processes are categorized into three categories, viz., physical, chemical, and biological, each having specific advantages depending on the wastewater characteristics [2] (Figure 2). Physical and chemical wastewater treatment processes are often used in conjunction with biological treatment to enhance the removal of pollutants and to meet discharge standards. These bioprocesses are relatively low-cost, efficient, and environmentally friendly in terms of treatment.

Physical treatment involves the separation of solids, liquids, and gases by using physical forces, such as gravity, filtration, sedimentation, flotation, and screening. Physical treatment methods are usually simple, low-cost, and effective for removing suspended solids, oil and grease, and some metals from wastewater. However, physical treatment methods cannot remove dissolved or colloidal pollutants, such as organic matter, nutrients, pathogens, and toxic substances. Sedimentation involves the settling of solid particles in a tank or basin by gravity, while filtration aids in solid particle removal by passing the wastewater through a porous medium, such as sand, gravel, or a membrane. Flotation aids in solid/liquid particle separation by injecting air bubbles into the wastewater and forming a floating layer of particles that can be skimmed off, whereas, in centrifugation, phase separation is carried out by spinning the wastewater at high speed and creating a centrifugal force.

Chemical treatment involves the addition of chemicals/agents to facilitate pollutants’ removal by altering their physical/chemical properties, such as pH, charge, solubility, and reactivity. Coagulation, flocculation, precipitation, ion exchange, neutralization, adsorption, and disinfection are performed in these treatment strategies using alum, lime, iron salts, polymers, chlorine, ozone, UV light, etc. [33,34]. Chemical treatment methods can be effective for removing dissolved or colloidal pollutants, such as organic matter, nutrients, pathogens, and toxic substances, from wastewater. However, chemical treatment methods can be expensive and complex and generate secondary wastes that require further treatment or disposal. Coagulation and flocculation involve coagulant and flocculant addition, respectively, to wastewater to form larger and heavier particles that can be settled/filtered more easily. Precipitation involves the addition of chemicals to wastewater to form insoluble compounds that can be settled or filtered out. Ion exchange occurs between wastewater and a resin/zeolite that can adsorb or release specific ions for treatment. The adjustment of the pH of wastewater by adding acids or bases leads to neutralization, making it more suitable for biological treatment or effluent discharge. Disinfection helps in the inactivation/destruction of pathogens in wastewater (*vibrio* and *mycobacterium*, *clostridium*, *salmonella*, *acinetobacter*, *aeromonas*, *pseudomonas*, etc.) by using chemicals such as chlorine, ozone, or hydrogen peroxide [14].

Biological treatment methods are based on the use of microorganisms to degrade or transform the organic matter and nutrients in the wastewater [25]. Some common biological treatment methods are aerobic, anaerobic, anoxic, and facultative processes, bioremediation, phytoremediation, and mycoremediation [35,36]. Aerobic treatment methods include activated sludge, trickling filters, rotating biological contactors, and oxidation ponds, which require oxygen for the breakdown of organic matter for wastewater treatment [37]. Anaerobic treatment methods use anaerobic digesters and up-flow anaerobic sludge blanket reactors, that operate in the absence of oxygen to treat wastewater and leads to methane production [29]. Anoxic treatment methods include denitrification filters and anoxic zones in activated sludge systems, using nitrate as an electron acceptor to reduce nitrate to nitrogen gas. Phytoremediation methods include the use of constructed wetlands, floating aquatic plants, and terrestrial plants to uptake, accumulate, or degrade pollutants in wastewater [38]. Biological treatment methods can be efficient, economical, and environmentally friendly for removing organic matter, nutrients, pathogens, and some toxic substances from wastewater [2,39]. However, biological treatment methods can be sensitive to environmental conditions, such as temperature, pH, oxygen, and toxicity, and require careful monitoring and control.

Biological wastewater treatment methods are advantageous in that they can treat a wide range of pollutants and nutrients, resulting in energy/chemical generation with efficient organic removal, thereby improving the wastewater quality and reducing the environmental impact of wastewater discharge [40]. The disadvantages lie in the large land area required and a long retention time, especially for aerobic treatment. These methods are sensitive to temperature, pH, toxicity, and shock loads, which can affect the performance and stability of the biological process [37]. Anaerobic treatment can produce excess sludge with odor problems, requiring further treatment prior to disposal. Challenges and opportunities for biological wastewater treatment include the optimization of process design and operation with single or integrated processes to achieve higher efficiency, lower cost, and lower energy consumption. Novel strategies with controlled microbial activity and microenvironments need to be developed for the recovery of nutrients, resources, and value-added products from wastewater and sludge to increase sustainability. Tuning diverse microbial communities with specific redox potentials could streamline biological wastewater treatment processes for the efficient removal of multiple pollutants (both conventional and emerging compounds), along with energy recovery and resource recovery/recycling to achieve a sustainable development nexus system [40].

In conclusion, physical, chemical, and biological wastewater treatments are important processes that can be used separately or in combination to treat different types of wastewaters and achieve different treatment goals. The selection of the most suitable treatment method depends on various factors, viz., wastewater characteristics, effluent quality, resource availability, environmental impacts, and economic feasibility. In most cases, a combination of physical, chemical, and biological treatment methods is used to achieve optimal results. Wastewater treatment is a vital process that can protect the environment and human health, conserve water resources, and recover valuable materials from wastewater.

## 3. Emerging Trends

A persistent problem related to the presence of toxic complexes/emerging pollutants/micropollutants in industrial waste/wastewater is their low degradability with biological treatment methods in spite of being environmentally friendly [33,41]. In this context, global research is emphasizing electrochemical interventions for the increased/improved degradability of industrial wastewater as a potential solution with respect to both electrochemical and bioelectrochemical processes [33,42]. Application orientation with specificity has resulted in the development of processes such as electrochemical oxidation, electrocoagulation, electro-flocculation, the electro-Fenton process, advanced oxidation processes, and bioelectrochemical treatment [33]. Individual or integrated systems have a broad scope and a greater impact on industrial wastewater treatment, addressing sustainability with water scarcity/purification problems [42,43,44]. This section intends to showcase the emerging and novel applications of upcoming and futuristic electrochemical processes that could be efficient in addressing industrial wastewater management.

### 3.1. Advanced Oxidation Processes

Advanced oxidation processes (AOPs) with chemical-based approaches are prominently used in wastewater treatment as primary processes [45,46]. But these processes have many limitations in terms of requiring proportional quantities of hydroxyl radicals and chemical reagents according to the presence of the wastewater pollutants to be scavenged, which makes the process non-feasible for handling huge loads of wastewater when operated as individual primary/secondary units, along with being economically infeasible [47]. In this context, futuristic AOP approaches integrated with other processes have gained significant importance, such as electro-Fenton processes, photo-Fenton processes, electrochemical oxidation, electro-flocculation, electro-flotation, photo-/electro-catalytic materials combined with UV systems (TiO_2_/UV or H_2_O_2_/UV), etc. [45,48]. These advanced integrative processes could increase the cumulative wastewater treatment with a stage-wise approach, reducing chemical/reagent usage along with lowering the costs for operational feasibility as compared to conventional AOPs [16,17,41]. Though electrochemical processes have benefits in industrial wastewater treatment, the need to optimize the operational parameters and electrode materials in specified combinations to design a reactor raises the estimated costs for application feasibility [10]. Hence, future research could focus on utilizing biological and renewable energy interventions in electrochemical processes for application to industrial wastewater treatment to decrease the overall environmental impact.

### 3.2. Electrochemical Methods

Conventional electrochemical processes have a wide scope of application in industrial/complex wastewater treatment, viz., textile dye, tannery, petroleum, food, paper and pulp, and oil and gas industries [49,50]. Electrocoagulation is a well-known process for the effective treatment of complex wastewater [34]. Its advantage lies in its capability to remove toxic contaminants, such as petroleum-derived hydrocarbons, organics/inorganics, salts/solids/oils, and heavy metals, with the cumulative advantages of coagulation, flotation, and electrochemistry, as these materials are otherwise difficult to remove via chemical/filtration treatment methods [51,52]. Its ability to treat wastewater without the addition of chemical inputs/coagulators is an advantage for toxin and odor removal, thereby reducing the overall operational costs and carbon footprints [49]. The electrolytic oxidation of anodic materials during the electrocoagulation process helps in the *in situ* generation of coagulants rather than external supplementation, resulting in wastewater treatment with very minimal generation of sludge [45,47]. Electrooxidation (EO) is an advanced oxidation process (AOP) used for wastewater treatment, particularly industrial effluents. In this technique, two electrodes (anode and cathode) connected to a power source generate strong oxidizing species. These species interact with contaminants, breaking them down into intermediates and eventually converting them into water and CO_2_. The process is effective against recalcitrant organic pollutants that conventional methods struggle to degrade. Electrode materials are crucial for effective process design with operational longevity, where cost, durability, and compactness with the application of low currents/voltages need to be considered [7,34,53,54]. The integration of these processes with tertiary and polishing systems can also further enhance the cumulative influence on industrial wastewater treatment with the consideration of multiple parameters for near-zero discharge [16].

### 3.3. Bioelectrochemical Remediation

**Figure 3:**
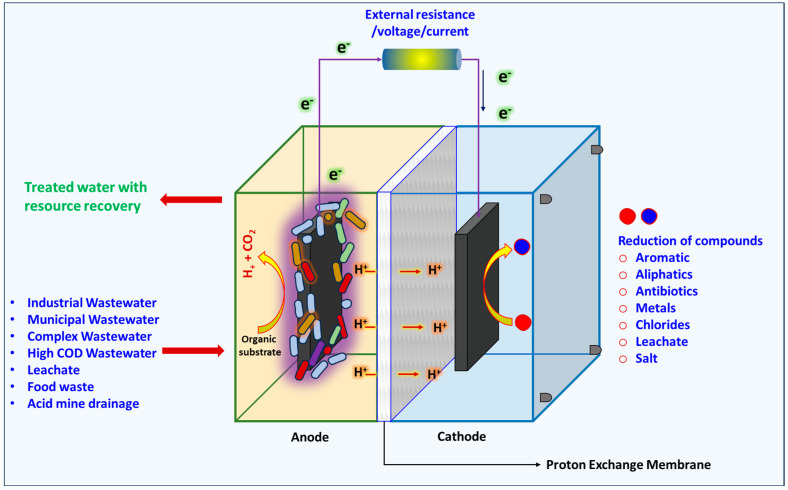
Bioelectrochemical systems and mechanisms involved in wastewater treatment with resource recovery.

In the context of addressing sustainability and the limitations of conventional electrochemical processes, the integration of microbial and electrochemical processes (bioelectrochemical treatment (BET)/electrochemical oxidation (EO) process) has arisen as a promising alternative for industrial wastewater treatment with near-zero carbon/micropollutant discharge [42,48,55,56]. Bioelectrochemical treatment processes (BET/EO/electrodeposition/biomineralization) have significantly influenced wastewater treatment because of their relatively high efficiency with integrated microbeelectrode interactions [20,22,43,56,57]. These integrated processes evaluate industrial wastewater treatment at multiple levels of assessment, with the influence of symbiotic reactions increasing the process efficiency. These processes involve microbial electrocatalysis, and the BET/EO process potentiality lies in its ability to degrade a spectrum of pollutants/contaminants/toxins in applications including wastewater treatment (BET/EO), salt removal (desalination), and metal removal/recovery (electrodeposition/biomineralization), taking advantage of the symbiotic reactions that take place in the hybridized biological and electrochemical system [19,58,59,60] (Figure 3). Anodic biofilm attachment and microbial community entities in relation to the synergy with the electrode play a crucial role in complex (pollutants/contaminants/toxins/metals) treatment by metabolically biocatalyzed electrochemical oxidation [61]. Anodic complex degradation in BET usually takes place by the mechanisms of direct anodic oxidation (DAO) and indirect anodic oxidation (IAO) processes [10,20]. DAO involves microbial metabolism utilizing simple organic molecules for electron (e^−^) and proton (H^+^) production, creating a gradient through their movement to the electrode surface and in situ biopotential generation [7]. During this electron transfer under a potential gradient, the e^−^ and H^+^ react with a water molecule for the production of primary oxidizing agents (nascent oxygen (O *) and hydroxyl (OH^−^) free radicals), which further bind to active sites on the anode surface, resulting in DAO, either alone or in conjunction with Cl^−^ ions in the wastewater [57,62]. These electrochemical interactions between the primary oxidants and wastewater complexes on the anode surface result in secondary oxidant production in the system. The IAO process usually involves the mediation of secondary oxidants (hydroxyl radicals (OH *), chlorine dioxide (ClO_2_), ozone (O_3_), hypochlorite (OCl^-^), and hydrogen peroxide (H_2_O_2_)), whose formation is proportional to the primary oxidant production [7,20,55]. Both the primary and secondary oxidants have a huge influence as mediators/redox intermediates during complex/industrial wastewater treatment. especially with respect to pollutants/contaminants/toxins, color removal, salt/TDS reduction, and metal removal/recovery [19,56,63,64].

#### Conducting Materials—Electron Sinks/Redox Shuttlers

The selection of electrode materials with optimal properties for biocompatibility is a challenge, and diverse conductive carbon/non-carbon materials are being used for pollutant/contaminant/toxin/metal removal in BETs [51,62,65]. The in situ/external biopotential generated/supplemented by the anode and cathode gradient and the material properties help in the removal of salts/TDS along with organic substrates (COD). The additional provision of an electrogenic environment (applied voltage/current) via the electrode materials in the BET system increases the treatment efficiency while reducing the hydraulic retention time (HRT) of operation. The electrode material properties best suited for increasing the adsorptive ability to enhance biofilm enrichment are a priority in establishing mutual microbe–electrode interactions [48,62,66]. The resultant symbiotic reactions enhance the effective electron exchange between the electroactive microorganisms and other pollutants/contaminants/toxins/metals, thereby triggering the redox reactions responsible for the degradation of complex pollutants with simultaneous bioelectricity generation [18,67,68]. The potential gradient developed in the microbe–material environment offers a variety of possible oxidative and reductive mechanisms and has opened new perspectives for bioelectrochemical remediation applications in the waste management domain. Electrochemically active bacteria (EABs) on the anode oxidize the substrate to release electrons by direct or indirect extracellular electron transfer (EET). EABs as cathodic catalysts enhance the cathode reduction rate, resulting in enhanced performance [57,63,69]. Electrochemical bioprocesses increase/regulate DIET between microbes for the effective treatment of complex wastewater. Different electrode conditions will influence the structure of the EAB community, which finally determines the performance of these processes. Realizing the potential of these processes requires a better understanding of the symbiotic microbe and electrode mechanisms (anode/cathode) for their practical implementation with economic/process feasibility in the waste management domain. Understanding this process with respect to the geographical sites for their specified boundary applications is critical for scale-up and transition at the real-field level.

### 3.4. Integrated Bioelectrochemical Remediation

Biological processes individually possess the capability of producing low-carbon/renewable energy/products depending on the microbial ability to metabolize various solid/liquid/gaseous substrates. Combined recyclable conductive carbon/non-carbon materials as hybrid processes, when integrated with biological processes, positively alter the mechanism’s ability to attain higher renewable energy/products/low carbon [70]. Biological processes are target-specific for product formation, along with byproducts/intermediates, depending on the individual process parameters, viz., anaerobic dark fermentation (biohydrogen), anaerobic digestion (biomethane), acidogenesis (short/medium-chain fatty acids), anoxygenesis (biopolymers), bioremediation (waste/wastewater treatment), CO_2_ sequestration (short/medium-chain alcohols), biogas upgradation (enhanced biomethane), biomineralization/electrodeposition (metal removal/recovery), solventogenesis (bioethanol/biobutanol), and composting (biofertilizer) [24,71,72]. Conductive materials (carbon-based (graphite, graphene, carbon cloth, carbon felt, activated carbon, etc.) and metal-based (stainless steel, titanium oxide, etc.) electrodes) provide biological systems with a potential gradient (in situ/applied), which could be an alternative to genetic manipulations for higher product/energy formation [21,73]. These non-genetic approaches induce increased electrogenic activity in microbes and effectively contribute to microbe–microbe/microbe–electrode interactions, increasing process efficiency [58]. In situ/applied energy during the bioelectrochemical process increases the electron transfer through the conduction band in a conductive material, which is used as a source of reducing equivalents by the electroactive bacteria to derive specific biobased products from solid/liquid/gaseous organic substrates. Hence, the application feasibility of these biohybrid systems is largely dependent on the conductive material properties [74]. The application feasibilities of conductive hybrid materials with respect to the individual biological processes against their product specificity are presented for consideration. A conductive material’s presence in biological processes creates an electrochemical environment that enhances the rate kinetics of reducing equivalents (electrons and protons), enabling the generation of the in situ biopotential necessary for the regulation of the electron flux to overcome the system losses [23,71,75]. The application feasibility for specific products depends on several factors, viz., the nature of the material, particle size, biocompatibility, surface area, adsorption ability, mechanical/tensile strength, longevity, etc., which govern the microbe–electrode interactions, biofilm stability, and electron flux, resulting in the overall system performance for catalyzing efficient redox reactions [76,77,78,79]. The biocompatibility of conductive materials is essential to establish improved intermediate metabolic and catalytic activities between the microbe and electrode, along with the biocatalyst’s adherence to the electrode surface. Microbial diversity enrichment is dependent on the type of conductive material/wastewater/substrate/targeted pollutant, where specific bacteria are enriched from a mixed microbiome in a gradual operation toward enhancing biobased products, viz., hydrogen, methane, short- and medium-chain carboxylic acids, alcohols, polymers, etc. [22,38]. Bioelectrochemical processes can be used as standalone processes or integrated as units into existing ETP/STPs for centralized/decentralized applications but have certain limitations in terms of treatment efficiency that need to be overcome [17]. Integrating/hybridizing these bioelectrochemical remediation strategies with non-genetic manipulations (conductive materials/in situ/applied potential) could improve the regulation of electron-flux-mediated microbial interactions for the increased treatment of industrial compounds (contaminants/pollutants/toxins/metals) [43,64,66]. These processes could be designed as membrane/membrane-less operations that can function effectively with all types of complex wastewater while augmenting the performance of activated sludge processes (ASPs), sequential batch processes (SBRs), anaerobic/aerobic/anoxic reactors, ecologically engineered systems (EESs), and constructed wetlands (CWs) with respect to treatment efficiency [31,72,80] (Table 1).

## 4. Sustainable Intervention

### 4.1. Resource Recovery

Despite the importance of conducting materials in effectively facilitating electron transfer, it is imperative to note that these materials play a pivotal role in determining the fate of biobased product formation [88]. Though the biocatalyst and the microenvironment dictate metabolic pathways and the corresponding biobased products, the role of electron flux is crucial in the carbon chain length, titer, and switching toward specific products’ formation, viz., short/medium-chain carboxylic acids (acidogenesis/carboxylate platform), short/medium-chain alcohols (solventogenesis), and biopolymers/bioplastics (anoxygenesis) [32,89,90,91]. The carboxylate platform/acidogenesis is comparatively a more advantageous bioprocess than others in terms of generating lower CO_2_ emissions compared to sugar platforms. Also, it facilitates the efficient utilization of diverse waste/wastewater/biomass streams, resulting in higher levels of short/medium-chain fatty acids along with the co-generation of biogas, biopolymers, and electricity production. The carboxylate platform is also a building block/substrate/precursor in several biological processes [89]. These have an application feasibility/scope in both biological and chemical industries. Platform chemicals and their derivatives are high-value chemicals and have significant importance in various sectors, viz., the food industry, research laboratories, pharmaceuticals, cosmetics/perfumes, polymers, fuel industries, etc. [24,92]. Platform chemicals include acetic acid, ethanol, methanol, butyric acid, caproic acid, lactic acid, acrylic acid, propionic acid, ethanol, butanol, succinic acid, citric acid, adipic acid, etc. [92,93]. Hence, the electrochemical integration of these biological processes using conductive materials and electron flux regulation is a necessity to enhance their productivity. The presence of electron-withdrawing groups on the surface of conducting materials effectively promotes electron transfer, thereby crucially contributing to surface-catalyzed bioelectrochemical reactions [10]. A porous and amenable surface morphology is crucial for biofilm formation, which encourages bioelectrochemical reduction reactions [77,88].

Nevertheless, it must be noted that electrophilic groups/charged groups on the surface of an electrode coupled with the aforesaid morphology will have a greater impact on the overall electron flux/process regulation [94]. The recent literature also focuses on a few carbon/non-carbon conductive materials having electrophilic surface groups, which could be effective in increasing the electron-holding capacity (capacitance) for the regulated electron charge–discharge between the microbe and materials. Capacitance, with respect to charge-holding/electron flux regulation and in situ/applied potential, could further channelize electron movement/utilization toward the redox acceptor/sink that aids in the specified product formation. Both carbon/non-carbon-based conductive materials have the capability of augmenting redox reactions by acting as an electron shuttler/promoter/reservoir. Although the literature illustrates that the utilization of several electrode materials plays a key function in bioelectrochemical redox reactions, the role of conductive materials in electron channeling is limited and deserves attention.

### 4.2. Circular Economy and Low-Carbon Footprints

Biological wastewater treatment utilizes microorganisms for organic pollutant removal/neutralization from wastewater for environmental restoration. A circular economy aims at maximizing resource recovery with increased carbon utilization efficiency by reusing, recycling, and recovering energy/chemicals from various sources toward zero discharge [16]. Biological wastewater treatment could be aligned with the principles of the circular economy and sustainable development goals (SDGs) by recovering valuable products from wastewater, viz., nutrients, biogas, biofuels, bioplastics, and biosorbents. Carbon capture and utilization can be enhanced by integrating conventional and advanced biological wastewater treatment systems, such as bioelectrochemical systems and anaerobic digestion. Advanced sludge treatment technologies, such as hydrothermal carbonization, pyrolysis, and gasification, also need to be focused on for low-carbon generation. Innovation with respect to energy- and resource-efficient treatment systems for reducing the environmental impact needs to be considered [1]. Integrated and advanced bioremediation strategies could contribute to the reduction in environmental pollutants by aligning with SDGs, such as Goal 3: Good Health and Well-being; Goal 6: Clean Water and Sanitation; Goal 11: Sustainable Cities and Communities; and Goal 13: Climate Action. Bioremediation approaches like chemotaxis, biostimulation, bioaugmentation, biofilm formation, the application of genetically engineered microorganisms, and advanced omics have been widely used. The microbial metabolic potential could be tremendously utilized for the sustainable degradation and remediation of environmental pollution, along with product generation. These strategies can help to transition wastewater treatment plants from being energy consumers to energy producers, and from being waste generators to resource providers, thus contributing to a more sustainable and resilient water sector.

## 5. Way Forward

Biological wastewater treatment has constraints and obstacles due to the fluctuating quality and quantity of wastewater and the toxicity of some molecules [95]. With the inter-dependence of anaerobic–aerobic treatment systems, considering the individual process advantages in designing an efficient treatment system that can simultaneously produce biogas and reduce the organic load can help in low-carbon generation. The combination of biological treatment with membrane filtration is an effective option to produce high-quality effluent and reduce sludge production. Biosorption with biomass/biochar adsorbents could be effective for removing heavy metals/dyes/other contaminants from wastewater. Biocatalysts with enzyme/microalgae utilization are effective in degrading or transforming recalcitrant pollutants, such as endocrine disruptors, pharmaceuticals, or textile dyes. Therefore, integrated strategies that combine biological treatment with other physical, chemical, or biotechnological methods could benefit in enhancing the performance and sustainability of wastewater treatment.

Futuristic emerging trends need to emphasize widening the application feasibility of these integrated bioprocesses. Hybridized strategies with genetic engineering and synthetic biology could be thoroughly aligned to enhance the capabilities and performance of microorganisms, such as improving their resistance, specificity, or productivity. Nanotechnology/nanomaterial applications could help to improve the properties and functions of membranes, adsorbents, or biocatalysts, such as increasing their selectivity, stability, or reusability [26]. The integration of artificial intelligence and machine learning to optimize the design, operation, and control of wastewater treatment systems, such as predicting the behavior, performance, or quality of systems, is widely recommended for the practical and economic viability of bioremediation strategies toward environmental sustainability [95]. Artificial intelligence is revolutionizing the wastewater treatment sector, presenting intriguing opportunities to enhance effectiveness, eco-friendliness, and robustness. Overall, integrated advancements in biological wastewater treatment could lead to the development of efficient, robust, and adaptable systems that can manage the increasing complexity and diversity of wastewater while recovering valuable energy/chemicals/resources to minimize the environmental impact and the economic cost of wastewater treatment.

## Figures and Tables

**Table 1:** Integration of various processes for wastewater treatment.

Wastewater	Treatment Process/Method	Treatment Efficiency	Reference
Vegetable oil factory	Ultrafiltration	COD removal: 91% TSS removal: 100%	[81]
Synthetic emulsified oily wastewater	Microfiltration	Organic pollutant removal: 95%	[82]
Landfill leachate	Integration of acidogenic and bioelectrochemical systems	COD removal: 71.21%	[83]
Metal finishing industry	Ultrafiltration integrated with reverse osmosis	Contaminant removal: 90–99%	[84]
Phenolic wastewater from paper mill industry	Ultrafiltration integrated with nanofiltration and reverse osmosis	COD removal: 95.5% Phenol removal: 94.9%	[85]
Paper and pulp wastewater	Bioelectrochemical treatment system	COD removal: 95% Color removal: 100%	[86]
Designed synthetic wastewater	Bioelectrochemical treatment system with PANi/CNT nanocomposite anode	COD removal: 80%	[75]
Glucose-based synthetic wastewater	Sono-bioreactor with 20 kHz: 2 W and 4 W	COD removal: >90%	[87]

## Abbreviations

ASP	Activated sludge process
AOPs	Advanced oxidation processes
BET	Bioelectrochemical treatment
ClO_2_	Chlorine dioxide
COD	Chemical oxygen demand
CWs	Constructed wetlands
DIET	Direct interspecies electron transfer
DAO	Direct anodic oxidation
EES	Ecologically engineered systems
EO	Electrochemical oxidation
EABs	Electrochemically active bacteria
EET	Extracellular electron transfer
HRT	Hydraulic retention time
H_2_O_2_	Hydrogen peroxide
OH *	Hydroxyl radicals
OCl^−^	Hypochlorite
IAO	Indirect anodic oxidation
IET	Interspecies electron transfer
O *	Nascent oxygen
O_3_	Ozone
POPs	Persistent organic pollutants
SBR	Sequential batch process
SDGs	Sustainable development goals
